# Effects of breastfeeding on children’s gut colonization with multidrug-resistant Enterobacterales in peri-urban Lima, Peru

**DOI:** 10.1080/19490976.2024.2309681

**Published:** 2024-02-01

**Authors:** Maya L. Nadimpalli, Luismarcelo Rojas Salvatierra, Subhra Chakraborty, Jenna M. Swarthout, Lilia Z. Cabrera, Amy J. Pickering, Maritza Calderon, Mayuko Saito, Robert H. Gilman, Monica J. Pajuelo

**Affiliations:** a Gangarosa Department of Environmental Health, Emory Rollins School of Public Health, Atlanta, GA, USA; b Stuart B. Levy Center for Integrated Management of Antimicrobial Resistance (Levy CIMAR), Tufts University, Boston, MA, USA; c Laboratorio de Microbiología Molecular, Facultad de Ciencias e Ingeniería, Universidad Peruana Cayetano Heredia, Lima, Peru; d Department of International Health, Johns Hopkins Bloomberg School of Public Health, Baltimore, MD, USA; e Department of Civil and Environmental Engineering, Tufts University, Medford, MA, USA; f Asociación Benéfica Proyectos en Informática, Salud, Medicina, y Agricultura (PRISMA), Lima, Peru; g Department of Civil and Environmental Engineering, University of California, Berkeley, CA, USA; h Blum Center for Developing Economies, University of California, Berkeley, CA, USA; i Department of Virology, Tohoku University Graduate School of Medicine, Sendai, Japan

**Keywords:** ESBL, breastfeeding, human milk, Peru, urban informal settlement, antibiotic resistance, infant health

## Abstract

Children living in low-resource settings are frequently gut-colonized with multidrug-resistant bacteria. We explored whether breastfeeding may protect against children’s incident gut colonization with extended-spectrum beta-lactamase-producing *Escherichia coli* (ESBL-*Ec*) and *Klebsiella*, *Enterobacter*, or *Citrobacter* spp. (ESBL-KEC). We screened 937 monthly stool samples collected from 112 children aged 1–16 months during a 2016–19 prospective cohort study of enteric infections in peri-urban Lima. We used 52,816 daily surveys to examine how exposures to breastfeeding in the 30 days prior to a stool sample were associated with children’s risks of incident gut-colonization, controlling for antibiotic use and other covariates. We sequenced 78 ESBL-*Ec* from 47 children to explore their diversity. Gut-colonization with ESBL-*Ec* was increasingly prevalent as children aged, approaching 75% by 16 months, while ESBL-KEC prevalence fluctuated between 18% and 36%. Through 6 months of age, exclusively providing human milk in the 30 days prior to a stool sample did not reduce children’s risk of incident gut-colonization with ESBL-*Ec* or ESBL-KEC. From 6 to 16 months of age, every 3 additional days of breastfeeding in the prior 30 days was associated with 6% lower risk of incident ESBL-*Ec* gut-colonization (95% CI: 0.90, 0.98, *p* = .003). No effects were observed on incident ESBL-KEC colonization. We detected highly diverse ESBL-*Ec* among children and few differences between children who were predominantly breastfed (mean age: 4.1 months) versus older children (10.8 months). Continued breastfeeding after 6 months conferred protection against children’s incident gut colonization with ESBL-*Ec* in this setting. Policies supporting continued breastfeeding should be considered in efforts to combat antibiotic resistance.

## Introduction

Infections caused by antibiotic-resistant bacteria result in more than 1.2 million deaths annually.^1^ Often, such infections are caused by bacteria that have previously colonized mucosal surfaces on a patient’s body, such as the digestive tract.^2,3^ In some middle-income countries where the burden of bacterial disease remains high, a substantial portion of young children are gut-colonized early in life with antibiotic-resistant bacteria of critical concern, including extended-spectrum beta-lactamase (ESBL)-producing Enterobacterales.^4–7^ In addition to causing infections that are more challenging and expensive to treat, ESBL-producing Enterobacterales are used as “indicator organisms” by the World Health Organization to gauge the magnitude and interconnectedness of antibiotic resistance between humans, animals, and the environment.^8^ Thus, public health interventions aimed at reducing antibiotic resistance in the community often assess changes in ESBL-producing Enterobacterales to gauge effectiveness. Recent studies have investigated if improvements in clean water and sanitation access,^9,10^ for example, may be effective at preventing children’s colonization with ESBL-producing Enterobacterales in low- and middle-income country (LMIC) settings. However, few studies have explored whether breastfeeding could confer such benefits.

Breastfeeding provides complete nutrition for young children and exclusive breastfeeding is recommended for the first six months of life. In addition to supporting healthy development, breastfeeding could protect children from acquiring drug-resistant bacteria or limit their abundance in the gut. First, breastfeeding limits oral exposures to diarrheagenic enteric pathogens that may be present in breast milk substitutes (e.g. evaporated or powdered cow’s milk) or the materials used to prepare or provide them (e.g. water, feeding bottles).^11,12^ Contamination of breastmilk substitutes and feeding implements may be especially likely in settings that lack access to clean water and refrigerators. Second, human milk oligosaccharides (HMOs) support the growth of commensal gut bacteria (*e.g*., *Bifidobacterium* spp.) that tend to harbor few antibiotic resistance genes and can outcompete exogenous pathogens, thereby hindering their establishment in the gut.^13^ HMOs and other components of breast milk (e.g. maternal antibodies, lactoferrin, and glycopeptides, HAMLET [human alpha-lactalbumin made lethal to tumor cells]) can also directly neutralize pathogens or increase their susceptibility to antibiotics.^14–17^ Third, observational studies from high-income countries^18,19^ suggest that breastfeeding reduces subsequent antibiotic use, which can otherwise disrupt the gut microbiome and increase risks of acquiring antibiotic-resistant bacteria.^20^ However, the relationship between breastfeeding and reduced antibiotic use has not been demonstrated in middle-income settings, where antibiotic use may be more common among children.^21^ To date, whether breastfeeding may protect children from becoming gut-colonized with ESBL-producing Enterobacterales has not been explored in middle-income countries, where the burden of community-acquired antibiotic resistance is highest.

Members of our team recently completed a two-year prospective cohort study of enteric infections in a peri-urban, low-resource community of Lima, Peru, which included weekly stool sample collection from children (starting during the first month of life) and daily surveillance of mothers’ breastfeeding practices. Here, we examined how exposures to breastfeeding over the first 16 months of life were associated with children’s incident gut colonization with ESBL-producing Enterobacterales, as well as the duration of colonization episodes. We also explored changes in the diversity of ESBL-producing *E. coli* colonizing children’s guts over time.

## Methods

### Sample selection

We used stool samples and near daily surveillance data that were collected during a recent prospective cohort study of childhood enteric infections in Villa El Salvador, Lima, Peru (NIH R01AI108695-01A1).^22^ Pregnant women were identified through an initial population screening in the community and through community health centers. Full-term babies were enrolled approximately within one month after birth from February 2016-May 2017, and surveyed through at least two years of age. The cohort study was approved by the Institutional Review Boards of Asociacion Benefica PRISMA, Universidad Peruana Cayetano Heredia (UPCH), and Johns Hopkins University. Written informed consent was obtained from caretakers of the infants for both study participation and the use of collected specimens for subsequent research.

Field staff visited infants every day except Sundays to collect survey data from the previous 24 hours. If field workers were unable to visit an infants’ home (e.g., day was Sunday, caretaker was not home), field workers collected survey data for the missing day at the time of the next visit. Basic child growth measurements were also collected each month. Socioeconomic variables, including presence of animals in the household, were collected at recruitment and once yearly after that.

Starting at recruitment and weekly thereafter, caregivers kept a freshly soiled diaper for field worker collection. Approximately 1 g of each stool specimen was transferred by sterile swab into Cary Blair Transport media, then stored at 4°C through transport to the UPCH laboratory. At UPCH, swabs were streaked on MacConkey agar plates to isolate Enterobacterales bacteria for multiple downstream analyses. After overnight incubation at 37°C, a swab was used to collect all plate growth. Swabs were inoculated in 1 mL of tryptic soy broth (TSB) supplemented with 10% glycerol (w/v), then frozen at −80°C at UPCH. These archived samples are hereafter referred to as “MacConkey screens.” MacConkey screens began to be collected and stored in December 2016 (10 months after enrollment began).

### Detection of ESBL-producing enterobacterales

The earliest MacConkey screens available for analysis were collected around 1 month of age. We initially tested MacConkey screens collected at or near 1, 3, 6, 9, 12, and 16 months of age for the presence of ESBL-producing Enterobacterales. Because we detected a spike in ESBL-Enterobacterales positivity around 6 months of age, we additionally screened samples (where available) from 4, 5, and 7 months, for up to 9 samples screened per child in total. Briefly, a loopful (~10 µL) of each MacConkey screen was streaked on CHROMagar ESBL^TM^ (CHROMagar, Paris, France), which is selective for ESBL-producing Enterobacterales and differential for *E. coli* (*Ec*; pink colonies) and *Klebsiella spp*., *Enterobacter spp*., *and Citrobacter spp*. (KEC; blue colonies). CHROMagar ESBL was prepared in house from dehydrated media; we used laboratory-confirmed *E. coli* and *K. pneumoniae* that harbored *bla*_CTX-M_ alleles as positive controls and ESBL-non-producing *E. coli* and *K. pneumoniae* strains as negative controls (courtesy of Dr. Pablo Tsukayama, UPCH). After overnight incubation at 37°C, we selected up to two colonies of each phenotype for archival in TSB + 10% glycerol. We phenotypically confirmed ESBL production using the double disk diffusion assay and the following BD BBL sensi-discs: amoxicillin with clavulanic acid (AMC-30), cefepime (FEP-30), cefotaxime (CTX-30), and ceftazidime (CAZ-30).

Our estimated lower limit of detection using this approach was 2 log_10_ CFU/g-wet feces. The lower limit of detection was determined by multiplying the lowest detectable number of colony forming units per plate (1) by the volume of each MacConkey screen that was plated onto CHROMagar ESBL (10 µL) and dividing by the total volume of each MacConkey screen (1000 µL); we assume that any viable ESBL-producing colony present in the original stool specimen (1 g) grew and was captured by the MacConkey screen in this estimate.

### Exposure definitions

At each daily survey visit, caretakers were asked if their baby was breastfed in the past 24 hours. If yes, the field worker recorded whether formula or complementary foods, which could have included cows’ milk or other breast milk substitutes provided in a bottle, were also provided. If a caretaker responded that a child was not breastfed in the past 24 hours, they were asked if the child was given formula or complementary foods. Of note, women in this setting did not utilize breast pumps; field workers observed that breast milk was provided exclusively via direct feeding. For timepoints up to six months of age, we considered the proportion of days in the 30 days prior to a stool sample that a child received only human milk. For timepoints after 6 months, we considered the proportion of days in the past 30 days that a child received any human milk.

Caretakers were also asked about children’s antibiotic use and diarrhea in the past 24 hours. If a child had consumed any medication, caretakers were asked to provide the packaging so the trained field worker could confirm it was an antibiotic. Because survey data were collected daily, this packaging was typically available. A new antibiotic course began when caretakers reported antibiotic consumption following two days of no exposure; a course ended when the child did not consume antibiotics for two consecutive days. Children were considered to have had diarrhea if they experienced ≥ 3 liquid or semiliquid stools in the past 24 hours. For children younger than 2 months, the definition was based on the caretaker’s assessment that the child had diarrhea.^23^ Diarrhea episodes were calculated the same way as antibiotic courses. We tabulated the number of antibiotic courses and diarrhea episodes per child-year, both overall and in the 30 days prior to a stool sample.

### Outcome definitions

We tabulated the prevalence of ESBL-*Ec* and ESBL-KEC colonization at each sampling point (between six and nine samples per child). Only colonies with morphology representative of *E. coli* (pink) or KEC (blue) on CHROMagar ESBL that were also confirmed to produce ESBLs via the double disk-diffusion assay contributed to these counts. We also tabulated the incidence of these outcomes at each sampling point. A gut-colonization event was incident, or new, if a child’s MacConkey screen was negative for the given outcome at the previously examined timepoint.

### Statistical analysis

For timepoints up to six months of age, we examined whether the proportion of days in the past 30 days that a child exclusively received breastmilk was associated with ESBL-*Ec* and ESBL-KEC incidence at each sampling point. For timepoints between 6 and 16 months of age, we examined whether the proportion of days in the past 30 days that a child received any breastmilk was associated with ESBL-*Ec* and ESBL-KEC incidence. We estimated relative risk (RR) effect measures using conditional Poisson regression models with a generalized estimating equation and an exchangeable correlation matrix to account for the non-independence of observations within the same children.

We identified covariates that others have found to be associated with breastfeeding practices and/or ESBL-producing Enterobacterales gut colonization and constructed a directed acyclic graph (DAG) to identify potential confounders of this association (Figure S1). For each potential confounder, we used conditional Poisson regression models to evaluate associations with ESBL-*Ec* and ESBL-KEC incidence in our study population (Supplementary Tables S1 and S2). Covariates that were associated with either outcome at *p* < 0.2 were included in our final models; these included: vaginal birth (yes or no), number of diarrhea episodes in the past 30 days, number of antibiotic courses in the past 30 days, and household poultry ownership (yes/no). Covariates that were found to be collinear were excluded.

As a secondary analysis, we examined whether exclusive breastfeeding was associated with length of gut-colonization episodes during the first 6 months. We restricted this analysis to the first 6 months as we regularly screened samples (1, 3, 4, 5, 6 months) during this timeframe and therefore could better estimate duration of gut-colonization (as detected by culture). We considered a gut-colonization episode to have ended when we observed one negative sample after a positive sample; the date an episode ended was identified as the midpoint between the date of the last positive sample and that of the first negative sample. Children who were newly colonized at the last sampling timepoint were not considered in this analysis as duration of gut-colonization could not be calculated. Children who continued to be gut-colonized at 6 months were censored. Per our definition, children could experience multiple gut-colonization episodes during this time frame. We assessed the association between the proportion of days in the first 6 months that a child received only breastmilk and the duration of ESBL-Ec and ESBL-KEC gut colonization episodes (in days) using Poisson regression models with a generalized estimating equation and an exchangeable correlation matrix to account for the non-independence of observations within the same children. We adjusted for vaginal birth (yes or no), number of diarrhea episodes and antibiotic courses in the first 6 months, and household poultry ownership (yes/no).

All analyses were conducted in R, version 4.0.3, RStudio (version 1.1.463). Statistical significance was defined by α = 0.05 and *p*-values are two-sided unless stated otherwise.

### Whole genome sequencing of ESBL-Ec

To investigate changes in the diversity of ESBL-*Ec* colonizing the guts of young children across time, we sequenced the genomes of ESBL-*Ec* isolated at timepoints when children were primarily breastfeeding (i.e., received only breastmilk for 90% of the past 30 days) (*n* = 31) and from timepoints where children had also been receiving complementary foods for the past two months (*n* = 47). Of the 78 isolates sequenced in total, we included two isolates from each of 31 children and one isolate each from 16 additional children. Species confirmation was conducted using API20E kits (bioMérieux, Paris, France) for a subset (*n* = 12) of isolates. Methods for DNA extraction, genome assembly and characterization, and core genome phylogenetic analysis are provided in the Supplementary Material.

We used logistic regression models with a generalized estimating equation and an exchangeable correlation matrix to account for the non-independence of observations within the same children to examine differences in phylogroup distribution, occurrence of antibiotic resistance genes (including ESBL gene alleles), multilocus-sequence types (MLSTs), and clonal complexes (CCs, *i.e*., groups of closely related strains thought to have evolved from a single founder, defined here as at least 5/7 shared MLST alleles) between the two timepoints. We only examined differences in phylogroups, antibiotic resistance genes, MLSTs, and CCs that were detected in at least 3 genomes. Multiple comparison correction was performed using the Benjamini–Hochberg method.^24^

## Results

### Participant characteristics

MacConkey screens were available starting at ≤2 months of age for 112 of 345 children participating in the original cohort study (Figure S2). Individual and household characteristics for these 112 children are described in Table 1. Approximately half of children were male, and 70% were delivered vaginally. Most children’s households had access to an indoor toilet (81%) and indoor water connection (78%) at the time of enrollment, although more than half of households experienced water cuts. Over 25% of households did not own a refrigerator. One-third of mothers had not completed high school. Household poultry rearing was rare; nearly 90% of households did not own poultry at enrollment nor one year later.

**Table 1.** Characteristics of 112 children participating in a prospective cohort study of enteric infections in Villa El Salvador, Lima, Peru, 2016–2019.

	*N* = 112
Male (%)	55 (49)
Birth mode (%)	
Vaginal	78 (70)
Cesarean section	33 (30)
Missing	1
Mean no. of antibiotic courses consumed/child-year (SD)	4.34 (2.8)
Mean no. of diarrhea episodes/100 child-month (SD)	6.71 (4.9)
Maternal education level (%)	
Less than high school	38 (30)
At least high school	74 (70)
Household size (%)	
7 or more	27 (24)
5 to 6	41 (36)
≤4	44 (40)
Owns a refrigerator (%)	83 (74)
Water source (%)	
Public tap	20 (18)
Indoor piped water	87 (78)
Other	5 (5)
Number of times water cuts experienced in past 2 months (%)	
None	48 (43)
At least twice	37 (33)
3 times or more	26 (23)
Toilet type (%)	
Indoor flush toilet	91 (81)
Pit latrine	19 (17)
Other	2 (2)
Household owns poultry (%)	
Neither at enrollment nor during Year 2 visit	88 (79)
Both visits	10 (9)
Household owns dogs (%)	
Neither at enrollment nor during Year 2 visit	50 (45)
Both visits	45 (40)
Household owns cats (%)	
Neither at enrollment nor during Year 2 visit	34 (30)
Both visits	53 (47)

Number and percent are presented for categorical variables. Mean and standard deviation (SD) are presented for continuous variables. Household characteristics were surveyed during an annual questionnaire, administered for the first time at enrollment. Data reported here were collected during the enrollment questionnaire unless otherwise noted.

Antibiotic use was frequent in this setting, as previously reported.^25^ Children included in this sub-study of enteric infections consumed 4.3 antibiotic courses/child-year on average (range: 0–11.4 courses/child-year). The average duration of a single antibiotic course was 5.3 days (standard deviation (SD): 2.6 days).

### Breastfeeding patterns

**Figure 1.**
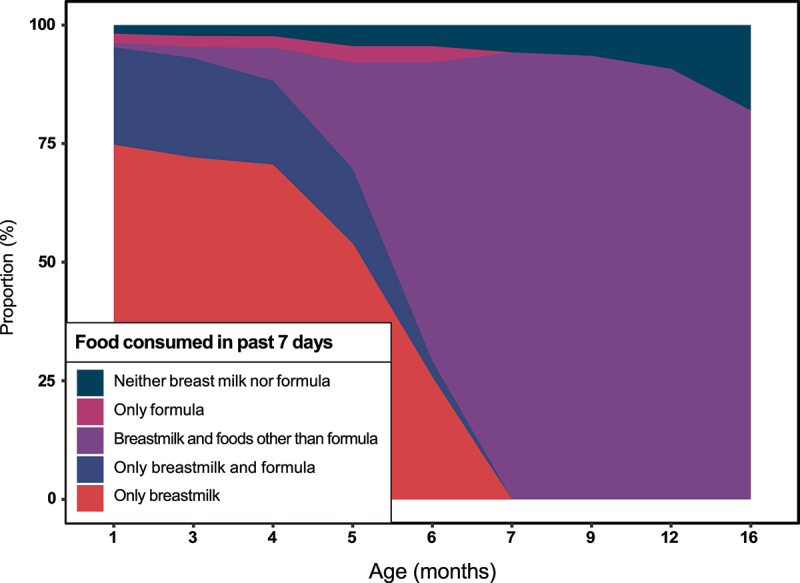
Changes in children’s exposures to breastmilk over the first 16 months of life, Lima, Peru, 2016–2019. Children’s feeding patterns were derived from 52,816 daily survey visits.

Most children in this sub-study of enteric infections were breastfed for at least some time (Figure 1). Leveraging 52,816 daily survey visits, we observed that less than 10% of children (7/112) were exclusively breastfed (per the World Health Organization’s definition of exclusive breastfeeding) for 6 months (median duration: 2.9 months, SD: 2.1), but more than 80% of children (95/112) continued receiving at least some breast milk through 16 months of age (Table 2). Most children began receiving foods other than breastmilk or formula around 5 months of age (median: 4.9 months, SD: 1.8 months). Only one child exclusively received formula in lieu of breastmilk, although more than half of children (63/112) received formula for at least some period.

**Table 2.** Early-life feeding patterns of 112 children participating in a prospective cohort study of enteric infections in Villa El Salvador, Lima, Peru, 2016–2019.

	*N* = 112
Exclusively breastfed for at least six months (%)	7 (6)
Median duration of exclusive breastfeeding in months (SD)	2.9 (2.1)
Consumed any formula (%)	63 (56)
Median age in months at introduction of formula (SD)	1.0 (1.7)
Number of children receiving any breastmilk at 16 months (%)	95 (85)
Median duration of any breastfeeding in months (SD)	16.4 (6.4)

Number and percent are presented for categorical variables. Median and standard deviation (SD) are presented for continuous variables.

### Prevalence and incidence of ESBL-Ec and ESBL-KEC

**Figure 2.**
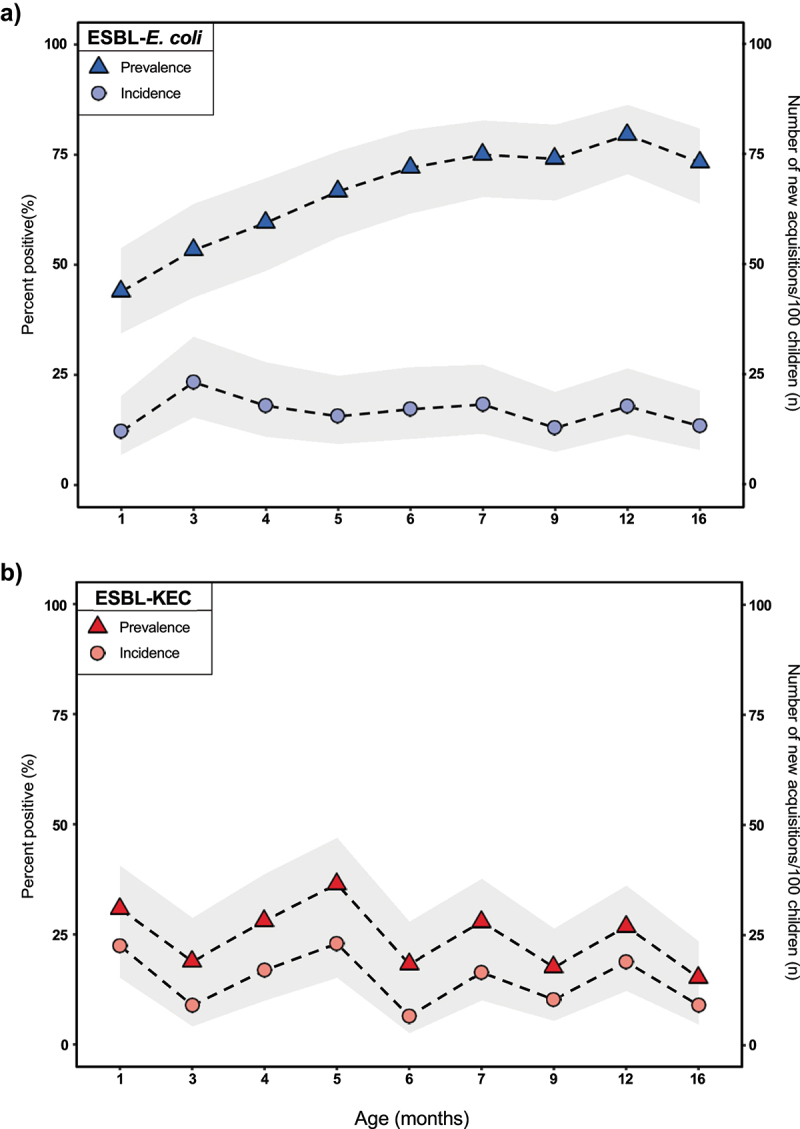
Gut colonization with ESBL-producing Enterobacterales over the first 16 months of life, Lima, Peru, 2016–2019. Changes in the mean point prevalence and incidence of a) ESBL-producing *Escherichia coli* and b) ESBL-producing *Klebsiella* spp., *Enterobacter* spp., or *Citrobacter* spp. Are depicted across specific age categories. Gray bands represent 95% confidence intervals around the mean.

*Note*: age (in months) was included as a continuous variable in all models. Age categories are depicted here for visualization purposes only: 1: ≥0.5 to <2months; 3: ≥2 to <3.5months; 4: ≥3.5 to 4.5months; 5: ≥4.5 to 5.5months; 6: ≥5.5 to 6.5months; 7: ≥6.5 to 8months; 9: ≥8 to <10.5months; 12: ≥10.5 to <14.5months; 16: ≥14.5months. For some children, two stool samples belonged to the same age bin, in this case, the earliest sample was chosen for depiction here.

We screened up to nine samples each from 112 children (minimum: 6, maximum: 9, median: 9) for ESBL-*Ec* and ESBL-KEC (Figure 2a, b). Of 937 stool samples screened in total, 66% were positive for ESBL-*Ec*. Over 40% of children with stool samples available at <2 months (47/107) were already gut colonized with ESBL-*Ec* at the first time point we screened, and prevalence significantly increased as children aged (*p* < .001 using a Cochrane–Armitage test for trend) to >70% by 16 months (82/112). The number of incident ESBL-*Ec* gut colonization events per age bin (as depicted in Figure 2a) ranged from 14 to 21 per 100 children. Eleven of 112 children did not experience any incident ESBL-*Ec* gut colonization events; 10 of these 11 were colonized at every timepoint we sampled while 1 of 11 was never colonized.

Twenty-four percent of stool samples (228/937) were positive for ESBL-KEC. Prevalence of ESBL-KEC colonization fluctuated between 15% and 36% per age bin (as depicted in Figure 2b) and unlike patterns observed with ESBL-*Ec*, tracked closely with incidence over time suggesting frequent gain and loss events. The number of incident ESBL-KEC gut colonization events per age bin ranged from 6 to 22 per 100 children. Twenty-six of 112 children did not experience any incident ESBL-KEC gut colonization events; 25 were never colonized while one was colonized throughout.

Nearly 70% of children (77/112) were co-colonized with ESBL-*Ec* and ESBL-KEC at least once during the study period.

### Associations between breastfeeding and incident gut-colonization with ESBL-Ec and ESBL-KEC

For timepoints earlier than 6 months, the proportion of the past 30 days that a child received only breastmilk was not associated with their risk of incident gut colonization with ESBL-*Ec* (Risk Ratio (RR): 0.98, 95% Confidence Interval (CI): 0.94, 1.02) nor ESBL-KEC (RR: 0.99, 95% CI: 0.95, 1.04) when controlling for child’s birth mode, household poultry ownership, and the number of antibiotic courses and diarrhea episodes that a child experienced during the 30 days prior (Table 3). The proportion of days that a child was exclusively breastfed over the first 6 months was also not associated with the duration of ESBL-*Ec* (β: 0.07, standard error (SE): 0.15, *p* = .60) or ESBL-KEC gut-colonization episodes (β: 0.13, SE: 0.08, *p* = .17) experienced during this time frame, when controlling for child’s birth mode, household poultry ownership, and the number of antibiotic courses and diarrhea episodes that a child experienced over the first 6 months.

**Table 3.** Associations between breastfeeding over the first 16 months of life and children’s incident gut colonization with extended-spectrum β-lactamase-producing enterobacterales, Lima, Peru, 2017–2019.

		ESBL-*Escherichia coli*			ESBL-*Klebsiella* spp., *Enterobacter* spp., *Citrobacter* spp.		
	Follow-up time (child-months)	Incident gut-colonization episodes per 100 child-months	Adj. Risk Ratio (95% CI)^a^	Pr (>|W|)	Incident gut-colonization episodes per 100 child-months	Adj. Risk Ratio (95% CI)^a^	Pr (>|W|)
1–6 months							
Percent of past 30 days child received *only* human milk^b^	471	23.1	0.98 (0.94, 1.02)	0.29	16.6	0.99 (0.95, 1.04)	0.78
>6–16 months							
Percent of past 30 days child received *any* human milk^b^	1037	7.7	0.94 (0.90, 0.98)	0.003	6.7	1.01 (0.95, 1.08)	0.74

Pr (>|W|) = Probability of the observed Wald test statistic if the null hypothesis were true.

a Adjusted for child age, vaginal birth (yes/no), number of diarrhea episodes in the past 30 days, number of antibiotic courses in the past 30 days, and household poultry ownership (yes/no).

b Modeled in increments of 10% (3 days).

For timepoints between 6 and 16 months of age, every three additional days of breastfeeding in the past 30 days was associated with 6% lower risk of incident gut colonization with ESBL-*Ec* (95% CI: 0.90, 0.98, *p* = .003) when controlling for child’s birth mode, household poultry ownership, and the number of antibiotic courses and diarrhea episodes that a child experienced during the 30 days prior (Table 3). This was equivalent to 60% reduced risk among children who received breastmilk every day in the 30 days prior to a stool sample. Continued breastfeeding after 6 months was not associated with lower risks of incident gut-colonization with ESBL-KEC (RR: 1.01, 95% CI: 0.95, 1.08).

### ESBL-Ec colonizing children’s guts

We sequenced 31 isolates collected when children were predominately breastfeeding (mean age: 4.1 months, SD: 1.4 months) and 47 isolates collected when children were still receiving breast milk but were also regularly consuming complementary foods (mean age: 10.8 months, SD: 1.9 months). Isolates from both timepoints were included for 31 children.

**Figure 3.**
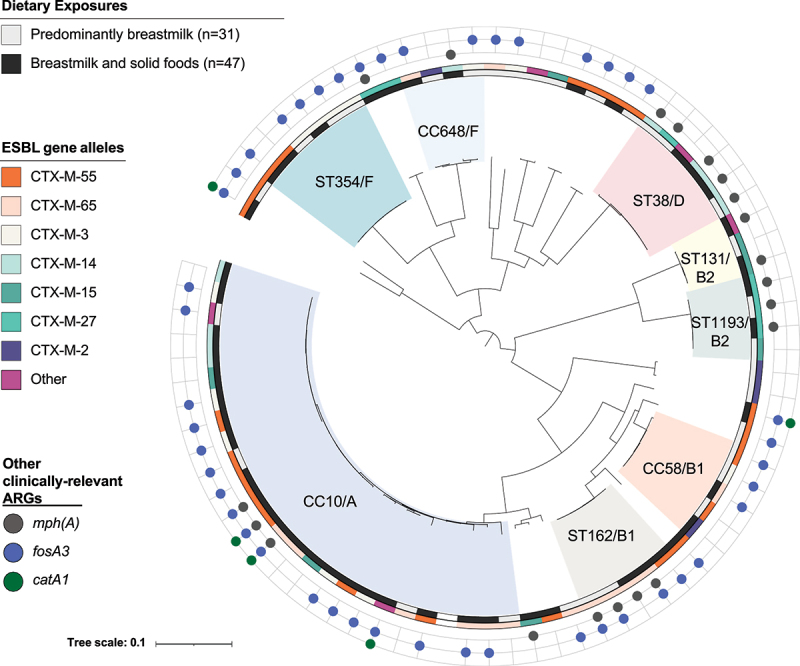
Core genome phylogenetic tree depicting the evolutionary relationships of 78 ESBL-producing *E. coli* from 47 children in Lima across two time points (predominantly breastfeeding versus breastfeeding while also eating complementary foods). We observed a high diversity of ESBL-*ec* and few differences in the occurrence of specific clonal complexes (CCs), sequence types (STs), phylogroups, ESBL alleles, or other antibiotic resistance genes (ARGs) across time points, suggesting the same ESBL-*ec* sequence types that were circulating among the broader community, in animals, and in the environment were also circulating within the household environment. Phylogroup designations A, B1, B2, or F are listed after highlighted CCs and STs.

We observed a wide diversity of ESBL-*Ec* clones among children in this setting (Figure 3, Table S3). Prevalent sequence types (STs) included STs often associated with extraintestinal infection, including ST131, ST1193, and ST38,^26,27^ as well as less commonly described clonal complexes (CCs) like CC648, recently reported in Spanish dogs.^28^ Most STs were just as commonly detected among children who were predominately breastfeeding versus those who were also consuming complementary foods, except for CC10 (comprising ST10 and single and double-locus variants), which was substantially more common among children consuming complementary foods and was the most common CC overall (fdr-corrected *p* < .05). Of the 31 children for whom isolates were sequenced at two distinct sampling points, only 1 child harbored the same ST at both time points (ST2732, phylogroup D). Most (40/78) ESBL-*Ec* isolates belonged to phylogroups A and B1 (Table S3), which are typically associated with commensal strains. The proportion of isolates belonging to phylogroups B1, B2, D, or F did not significantly change as children aged, but strains belonging to phylogroup A were significantly more common among children consuming complementary foods (fdr-corrected *p* < .05), likely driven by the increased proportion of CC10 strains.

As expected given their phenotype, all isolates harbored ESBL genes, with *bla*_CTX-M-55_ (23/78), *bla*_CTX-M-65_ (15/78), and *bla*_CTX-M-14_ (8/78) being the most common. There was no difference in the prevalence of any ESBL gene allele between timepoints, although *bla*_CTX-M-14b_ (*n* = 1) and *bla*_SHV-12_ (*n* = 1) were only observed among *E. coli* from children receiving complementary food. In addition to ESBL alleles, all isolates harbored additional resistance genes, most commonly *mdfA* (78/78) encoding a multidrug resistance efflux pump, *fosA3* (45/78) encoding fosfomycin resistance, *sul2* (42/78) encoding sulfonamide resistance, and *strB* (40/78) encoding streptogramin resistance. The *catA1* gene, which confers resistance to chloramphenicol, was exclusively detected among *E. coli* recovered from children consuming complementary foods (5/47). Genes conferring resistance to clinically important antibiotics other than third-generation cephalosporins, including *mph(A)* (conferring resistance to azithromycin) (20/78) and *qnrB19* (conferring low-level quinolone resistance) (15/78), were also observed among children’s isolates, although gene-mediated resistance to colistin and carbapenems were not detected. Other than the *catA1* gene, there was no difference in the occurrence of any other antibiotic resistance gene among *E. coli* recovered from predominately breastfeeding children (*n* = 31) versus children also consuming complementary foods (*n* = 47), after correcting for multiple comparisons.

## Discussion

In this cohort of 112 children living in a low-resource community of Lima, Peru, we leveraged over 50,000 survey observations to identify that continued breastfeeding after 6 months of age was associated with decreased risks of incident gut-colonization with ESBL-producing *Escherichia coli*, independent of children’s antibiotic use. The World Health Organization currently recommends continued breastfeeding up to 2 years of age to improve child nutrition and prevent illness, as well as to protect maternal health and well-being.^29^ In settings that lack consistent access to clean water and refrigeration, and where children are frequently exposed to fecal bacteria in their household environments, our findings suggest that continued breastfeeding after 6 months could protect children from incident gut-colonization with ESBL-*Ec*, which have been classified as a “serious” threat to human health by the United States Centers for Disease Control and Prevention.^30^

The specific mechanisms by which breastfeeding protects against children’s gut colonization with ESBL-producing Enterobacterales later in life requires further research, especially since exclusive breastfeeding during the first 6 months did not reduce children’s risk of incident gut colonization with ESBL-producing Enterobacterales nor was associated with shorter gut colonization episodes. In this setting, any protective effects of breastfeeding in the first 6 months may have been diluted by the common provision of formula and other breast milk substitutes. Breast milk substitutes like formula and evaporated or powdered cow’s milk are often prepared with water, which may be contaminated, and provided via bottles, which are frequently contaminated with fecal bacteria.^12^ Whether continued breastfeeding after 6 months was protective because it reduced children’s oral exposures to ESBL-producing Enterobacterales, or because it altered the gut microbiome environment in such a way that the proliferation of these bacteria or the resistance genes they harbored was inhibited, remains unclear. To our knowledge, this is the first report from a LMIC setting of the potential role of breastfeeding in protecting children against incident ESBL-*Ec* gut colonization.

Although continued breastfeeding after 6 months was associated with reduced risk of incident ESBL-*Ec* gut colonization, the prevalence of ESBL-*Ec* was already high at <2 months (44%) and increased to nearly 75% by 16 months. Gut-colonization studies from LMICs indicate dynamic microbial competition following ESBL-*Ec* acquisition, with new multidrug-resistant strains continually being acquired and partially displacing previously acquired ESBL-*Ec* and resident gut strains.^31,32^ Our whole genome sequencing analysis revealed a wide diversity of ESBL-*Ec* gut colonizing young children in this setting, including sequence types previously reported in animals and the environment in Bolivia, rural Peru, and Ecuador,^33–38^ as well as in market chickens sold in the same community (specifically, CC10, CC58, CC648, ST744, ST354, ST1196).^39^ MLSTs reported in livestock and bats in the Lima region during 2015–2018 were also detected among children in this study.^40^ We observed almost no similarity within children across time, suggesting that children were either gut-colonized with multiple ESBL-*Ec* sequence types simultaneously (in which case persistent colonization may have been missed, as we only sequenced one isolate per sample), or that predominant gut ESBL-*Ec* MLSTs shifted as children aged. Children were just as commonly colonized with most of the ESBL-*Ec* MLSTs we detected regardless of whether they were predominantly breastfeeding, indicating that the same ESBL-*Ec* MLSTs circulating among the broader community, animals, and the environment are also circulating within the household environment. While continued breastfeeding after 6 months might confer protection against incident colonization with ESBL-*Ec*, major reductions in the infectious disease burden and in human and animal antibiotic use will still be necessary to effectively reduce ESBL-producing Enterobacterales prevalence in this setting.

To our knowledge, this is the first report of ESBL-*Ec* and ESBL-KEC gut colonization rates among children living in a peri-urban setting in South America.^41^ Like other studies conducted in LMICs, we find that ESBL-*Ec* and ESBL-KEC colonization rates were substantially higher than reported among children in Western settings.^42,43^ The prevalence of ESBL-*Ec* gut colonization among children in this setting was similar to rates recently described among infants in Bangladesh.^4^ Notably, prevalence of ESBL-*Ec* gut colonization nearly doubled from age 1–16 months. This increase over time could indicate that the household environment is an important source of exposure to ESBL-*Ec*, particularly as children grow and increasingly interact with their immediate surroundings.

*E. coli* CC10 was the only clonal complex found to be significantly more common once children began eating complementary foods, compared to timepoints where they were predominately receiving breastmilk. *E. coli* CC10 is detected in humans, animals, and the environment and is one of the most common ESBL-*Ec* clonal complexes in several surveys of retail meats.^44–46^ We also observed that *catA1*, which confers resistance to chloramphenicol, was significantly more common among ESBL-*Ec* from children eating complementary foods. Chloramphenicol is rarely used in human medicine in Peru, but chloramphenicol analogs, namely florfenicol, are extensively used in industrial poultry production.^39^ Resistance to chloramphenicol and its derivatives has been proposed as a marker of chicken-origin in Peru and other countries where chloramphenicol is rarely used in human medicine but frequently in chicken production, although other genomic elements (i.e., *floR*) have previously been implicated.^39,44^ Per-capita chicken consumption is high in Peru^39^ and chicken products are commonly introduced to children early in life. Food consumption could be a unique source of CC10 ESBL-*Ec* or the *catA1* gene in this setting, or children could be acquiring these strains from other sources that they are only exposed to after 10 months of age.

The predominant ESBL allele among ESBL-*Ec* from children was *bla*_CTX-M-55_ (24/78), which has been widely reported among bacteria in Peru and neighboring countries in the past 10 years including among livestock,^40,47^ wild animals such as dogs,^34^ bats,^40^ and condors,^48^ healthy children,^34^ and in enteric infections.^47^ The second most prevalent ESBL allele was *bla*_CTX-M-65_ (15/78) which is commonly reported in *Salmonella enterica* serovar Infantis from animals and animal products in the region,^49–52^ although CTX-M-65-producing Enterobacterales causing bacteremia and diarrhea have also been reported among Peruvian children.^49,53,54^ Interestingly, both *bla*_CTX-M-55_ and *bla*_CTX-M-65_ are believed to have evolved in Asia.^55,56^ Our findings confirm that *bla*_CTX-M-55_ and *bla*_CTX-M-65_ are now widespread in the peri-urban community setting in Peru. Other common ESBL alleles that we observed include *bla*_CTX-M-3_ (12/78), *bla*_CTX-M-14_ (9/78), and *bla*_CTX-M-15_ (7/78), all of which have been reported among clinical infections in Peru and neighboring countries.^47,53,57^

A unique and important strength of this study was the ability to delineate children’s breastfeeding patterns by leveraging survey data from over 52,816 daily surveillance visits. Other studies of breastfeeding patterns on children’s antibiotic use or acquisition of multidrug-resistant bacteria have classified exposures based on 1- to 3-month recall periods,^18,58^ or up to 5 years after weaning.^19^ Daily surveillance data are much less susceptible to recall bias and uniquely allowed us to examine the effects of recent exposures to breastfeeding over time. Unlike in previous studies that have been conducted in high-income settings, continued breastfeeding through at least one year was common in this study population and allowed us to explore the association between this exposure and children’s incident gut-colonization with ESBL-*Ec* and ESBL-KEC. Our finding that continued breastfeeding after 6 months was highly protective against incident ESBL-*Ec* gut colonization has important implications for ongoing efforts to mitigate global antibiotic resistance.

We used culture-based methods to detect ESBL-producing Enterobacterales, which are limited by a high lower limit of detection (estimated at 2 log10 CFU/g wet feces in this study). It is possible that samples that were culture negative for ESBL-*Ec* and ESBL-KEC contained these bacteria at concentrations below the limit of detection, and thus incident gut-colonization episodes, rather than representing new acquisitions, in some instances may represent blooms of preexisting ESBL-producing species above the limit of detection. Culture-based methods remain the only way to cost-effectively detect bacterial species linked to a phenotype of interest; PCR methods which are orders of magnitude more sensitive could be used to quantify *E. coli*, *Klebsiella* spp., *Citrobacter* spp., or *Enterobacter* spp. in feces but not the proportion that are ESBL-producing, or conversely, to detect EBSL genes but not the bacteria that harbor them. Even if incident gut-colonization episodes do not represent new acquisitions, but rather fluctuations of preexisting species above the limit of detection, we believe our finding that continued breastfeeding reduces such episodes is clinically relevant since higher concentrations of multidrug-resistant Enterobacterales in the gut are associated with elevated risk of subsequent infection.^2,59^ Thus, any exposure that reduces or prevents blooms of ESBL-producing Enterobacterales in the gut may be beneficial for human health.

This study had other limitations. First, stool samples were not collected from mothers and thus it was impossible to ascertain if ESBL-*Ec* or ESBL-KEC detected in children’s first stool samples were seeded during birth or were new acquisitions. Second, we only screened up to two isolates per sample for phenotypic ESBL production and only selected up to one *E. coli* isolate for whole genome sequencing; thus, we may have underestimated ESBL-*Ec* and ESBL-KEC prevalence and ESBL-*Ec* diversity. Additional sequencing would have also helped distinguish if children who were continuously colonized with ESBL-*Ec* were colonized with the same strains over time or were continually acquiring new strains; we were unable to investigate strain-level dynamics using our culture-based approach and thus may have underestimated incident colonization events. Finally, because several children did not experience incident ESBL-*Ec* or ESBL-KEC colonization events during the study period, we had limited power to detect several associations of interest.

## Conclusion

This study examined whether breastfeeding, which is universally recommended for children less than 2 years of age, might also reduce children’s risk of acquiring ESBL-producing Enterobacterales in a peri-urban low resource community in Peru. Because we used daily surveillance data collected over the first 16 months of life, we were able to classify children’s feeding practices with a high degree of confidence. Our findings suggest that continuing to breastfeed after 6 months, even after children have begun to consume other foods, confers significant protection against children’s incident gut colonization with ESBL-*Ec*. Overall, policies that support continued breastfeeding after 6 months while ensuring mothers’ access to education and economic opportunities may be a useful tool in global efforts to curb the spread of antimicrobial resistance.

## Supplementary Material

SuppMaterial_Breastfeeding_AMR_Peru_2Dec2023.docx

## Data Availability

Short-read data are available in NCBI’s Sequence Read Archive (https://www.ncbi.nlm.nih.gov/sra) under BioProject number PRJNA821865. Other datasets analyzed here are available from the corresponding author upon reasonable request.
