# Supplementary material for: Effects of breastfeeding on children’s gut colonization with multidrug-resistant Enterobacterales in peri-urban Lima, Peru
Source: Gut Microbes. 2024 Feb 1;16(1):2309681. doi: 10.1080/19490976.2024.2309681 (PMC10841006; doi:10.1080/19490976.2024.2309681)
Supplement: SuppMaterial_Breastfeeding_AMR_Peru_2Dec2023.docx [file KGMI_A_2309681_SM9984.docx]

**Effects of breastfeeding on children's incident gut-colonization with multidrug-resistant Enterobacterales in peri-urban Lima, Peru**

Maya L. Nadimpalli, Luismarcelo Rojas Salvatierra, Subhra Chakraborty, Jenna M. Swarthout, Lilia Z. Cabrera, Amy J. Pickering, Maritza Calderon, Mayuko Saito, Robert H. Gilman, and Monica J. Pajuelo

**Appendix**

| **List of Tables** | **Page** |
| --- | --- |
| Table S1: Potential risk factors for children’s (n=112) incident gut-colonization with ESBL-producing *Escherichia coli* in Lima, Peru. | 4 |
| Table S2: Potential risk factors for children’s (n=112) incident gut-colonization with ESBL-producing *Klebsiella* spp., *Enterobacter* spp., or *Citrobacter* spp. (KEC) in Lima, Peru. | 5 |
| Table S3: Accession numbers for short-read whole genome sequence files of 78 ESBL-producing *E. coli* from children in Lima, Peru | 6-8 |

| **List of Figures** |  |
| --- | --- |
| Figure S1: Directed acyclic graph for the association between breastfeeding and incident gut colonization with ESBL-producing Enterobacterales | 9 |
| Figure S2: Participant flow chart outlining the previously collected samples and paired metadata included in this analysis | 10 |
|  |  |

**Methods**

Whole genome sequencing of ESBL-*Ec*

Bacterial DNA was extracted with the GeneJet Genomic DNA Purification kit (ThermoFisher) and sent to Tufts University’s Genomics Core for library preparation using the Nextera XT DNA Library Preparation Kit (Illumina) and paired-end, 150 bp sequencing using the NextSeq 550 platform. Short-read data are available in NCBI’s Sequence Read Archive under BioProject number PRJNA821865. Raw reads were trimmed using Trimmomatic v0.39 (1) and then assembled and scaffolded with SPAdes v3.14.1 (2). After quality checks using QUAST v.5.0.2 (3), we used ABRicate version v1.0.1 (https://github.com/tseemann/abricate) to search the ResFinder database v.4.1.3 for antibiotic resistance genes (100% identity threshold for beta-lactamase genes; 90% identity threshold for all other genes) (4). We used MLST v.2.19.0 (https://github.com/tseemann/mlst) to assign a multilocus-sequence type (MLST) based on the Achtman scheme and EzClermont v.0.6.3(5) to assign a Clermont phylotype (A, B1, B2, C, D, E, F, and clade I). Novel MLSTs were assigned using Enterobase (https://enterobase.warwick.ac.uk/). We identified core genome single nucleotide polymorphisms and indels using Snippy 4.6.0 (https://github.com/tseemann/snippy) and an *E. coli* ST10 genome (GenBank accession number: CP011113) as a reference. The full alignment was cleaned using the snippy-clean_full_aln function and filtered for recombination using default settings with Gubbins v.3.1.6 (6), resulting in an alignment of 245,139 sites. Variable positions present in all strains were then identified with SNP-sites v2.5.1 (7). The final alignment consisted of 4,728 SNP positions. We used FastTree v2.1 (8) to generate a maximum-likelihood phylogenetic tree under a GTR (generalized time-reversible) nucleotide substitution model with default settings. The final tree was visualized and annotated using ITOL version 6.5.2 (<https://itol.embl.de>).

**References**

1. Bolger AM, Lohse M, Usadel B. Trimmomatic: a flexible trimmer for Illumina sequence data. Bioinformatics. 2014 Aug 1;30(15):2114–20.

2. Bankevich A, Nurk S, Antipov D, Gurevich AA, Dvorkin M, Kulikov AS, et al. SPAdes: a new genome assembly algorithm and its applications to single-cell sequencing. J Comput Biol. 2012 May;19(5):455–77.

3. Gurevich A, Saveliev V, Vyahhi N, Tesler G. QUAST: quality assessment tool for genome assemblies. Bioinformatics. 2013 Apr 15;29(8):1072–5.

4. Bortolaia V, Kaas RS, Ruppe E, Roberts MC, Schwarz S, Cattoir V, et al. ResFinder 4.0 for predictions of phenotypes from genotypes. Journal of Antimicrobial Chemotherapy. 2020 Dec 1;75(12):3491–500.

5. Waters NR, Abram F, Brennan F, Holmes A, Pritchard L. Easy phylotyping of Escherichia coli via the EzClermont web app and command-line tool. Access Microbiol. 2020;2(9):acmi000143.

6. Croucher NJ, Page AJ, Connor TR, Delaney AJ, Keane JA, Bentley SD, et al. Rapid phylogenetic analysis of large samples of recombinant bacterial whole genome sequences using Gubbins. Nucleic Acids Research. 2015 Feb 18;43(3):e15–e15.

7. Page AJ, Taylor B, Delaney AJ, Soares J, Seemann T, Keane JA, et al. SNP-sites: rapid efficient extraction of SNPs from multi-FASTA alignments. Microb Genom. 2016 Apr;2(4):e000056.

8. Price MN, Dehal PS, Arkin AP. FastTree 2 – Approximately Maximum-Likelihood Trees for Large Alignments. Poon AFY, editor. PLoS ONE. 2010 Mar 10;5(3):e9490.

**Table S1**. Potential risk factors for children’s (n=112) incident gut-colonization with ESBL-producing *Escherichia coli* in Lima, Peru.

|  | Follow-up time  (child-months) | Incident gut-colonization episodes per 100 child-months | Risk Ratio (95% CI) | p-value |
| --- | --- | --- | --- | --- |
| Sex |  |  |  |  |
| Male | 813 | 10.8 | Reference |  |
| Female | 845 | 11.9 | 1.1 (0.94,1.30) | 0.25 |
| Delivery mode |  |  |  |  |
| Vaginal | 1165 | 10.7 | Reference |  |
| Cesarean section | 467 | 13.3 | 1.25 (1.04, 1.49) | **0.02** |
| Maternal education |  |  |  |  |
| Less than high school | 563 | 11 | Reference |  |
| At least high school | 1095 | 11.6 | 1.07 (0.90, 1.28) | 0.44 |
| Household PPI |  |  |  |  |
| 1^st^ quartile | 266 | 12.8 | Reference |  |
| 2^nd^ quartile | 457 | 11.6 | 1.02 (0.78, 1.33) | 0.88 |
| 3^rd^ quartile | 350 | 11.4 | 1.00 (0.76, 1.31) | 0.98 |
| 4^th^ quartile | 586 | 10.4 | 0.93 (0.71, 1.22) | 0.59 |
| Household owns chickens at time of enrollment |  |  |  |  |
| No | 1390 | 11.4 | Reference |  |
| Yes | 269 | 11.5 | 1.05 (0.86, 1.29) | 0.62 |
| Number of antibiotic courses/child-year |  |  |  |  |
| 0 | 734 | 12.2 | Reference |  |
| 1-3 | 452 | 12.2 | 1.01 (0.68, 1.51) | 0.95 |
| >3 | 1133 | 11 | 0.88 (0.59, 1.30) | 0.51 |
| Number of diarrhea episodes/child-month |  |  |  |  |
| 0 | 59 | 15.2 | Reference |  |
| 1-5 | 783 | 11.9 | 0.74 (0.59, 0.93) | **0.01** |
| >5 | 817 | 10.7 | 0.67 (0.53, 0.84) | **0.00** |

**Table S2.** Potential risk factors for children’s (n=112) incident gut-colonization with ESBL-producing *Klebsiella* spp.*, Enterobacter* spp.*, or Citrobacter* spp. (KEC) in Lima, Peru.

|  | Follow-up time (child-months) | Incident gut-colonization episodes per 100 child-months | Risk Ratio (95% CI) | p-value |
| --- | --- | --- | --- | --- |
| Sex |  |  |  |  |
| Male | 813 | 9.22 | Reference |  |
| Female | 845 | 8.52 | 0.91 (0.71,1.18) | 0.48 |
| Delivery mode |  |  |  |  |
| Vaginal | 1165 | 8.41 | Reference |  |
| Cesarean section | 467 | 10.3 | 1.20 (0.91, 1.56) | **0.19** |
| Maternal education |  |  |  |  |
| Less than high school | 563 | 7.99 | Reference |  |
| At least high school | 1095 | 9.31 | 1.13 (0.86, 1.48) | 0.40 |
| Household PPI |  |  |  |  |
| 1^st^ quartile | 266 | 9.79 | Reference |  |
| 2^nd^ quartile | 457 | 9.62 | 0.99 (0.68, 1.43) | 0.95 |
| 3^rd^ quartile | 350 | 8.86 | 0.94 (0.60, 1.50) | 0.81 |
| 4^th^ quartile | 586 | 7.68 | 0.90 (0.61, 1.33) | 0.59 |
| Household owns chickens at time of enrollment |  |  |  |  |
| No | 1390 | 8.49 | Reference |  |
| Yes | 269 | 10.79 | 1.34 (1.00, 1.79) | **0.05** |
| Number of antibiotic courses/child-year |  |  |  |  |
| 0 | 734 | 5.44 | Reference |  |
| 1-3 | 452 | 7.53 | 1.38 (0.60, 3.15) | 0.45 |
| >3 | 1133 | 9.62 | 1.76 (0.81, 3.83) | **0.16** |
| Number of diarrhea episodes/child-month |  |  |  |  |
| 0 | 59 | 6.78 | Reference |  |
| 1-5 | 783 | 8.94 | 1.29 (0.61, 2.74) | 0.51 |
| >5 | 817 | 8.94 | 1.32 (0.62, 2.82) | 0.47 |

**Table S3**. Accession numbers for short-read whole genome sequence files of 78 ESBL-producing *E. coli* from children in Lima, Peru.

| Accession Number | Child  ID | Collection  Date | Age  (months) | Feeding Pattern | ST | Phylotype | ESBL Gene Type |
| --- | --- | --- | --- | --- | --- | --- | --- |
| SAMN27150387 | 1 | 2017-02-27 | 4.4 | BM | ST354 | F | blaCTX.M.3 |
| SAMN27150388 | 3 | 2017-05-02 | 5.5 | BM | ST617 | A | blaCTX.M.55 |
| SAMN27150389 | 4 | 2017-01-16 | 1.4 | BM | ST1163 | G | blaCTX.M.55 |
| SAMN27150390 | 5 | 2017-02-20 | 2.8 | BM | ST218 | A | blaCTX.M.65 |
| SAMN27150391 | 6 | 2017-03-08 | 3.2 | BM | ST12392 | U/cryptic | blaCTX.M.65 |
| SAMN27150392 | 7 | 2017-03-07 | 2.8 | BM | ST349 | D | blaCTX.M.3 |
| SAMN27150393 | 9 | 2017-06-05 | 6.0 | BM | ST973 | D | blaCTX.M.65 |
| SAMN27150394 | 10 | 2017-05-30 | 5.7 | BM | ST58 | B1 | blaCTX.M.55 |
| SAMN27150395 | 11 | 2017-03-16 | 2.9 | BM | ST38 | D | blaCTX.M.14 |
| SAMN27150396 | 12 | 2017-06-04 | 5.8 | BM | ST1193 | B2 | blaCTX.M.27 |
| SAMN27150397 | 14 | 2017-04-04 | 3.2 | BM | ST448 | B1 | blaCTX.M.55 |
| SAMN27150398 | 15 | 2017-06-05 | 5.3 | BM | ST2732 | D | blaCTX.M.55 |
| SAMN27150399 | 16 | 2017-06-13 | 6.0 | BM | ST354 | F | blaCTX.M.3 |
| SAMN27150400 | 17 | 2017-05-23 | 5.2 | BM | ST648 | F | blaCTX.M.3 |
| SAMN27150401 | 18 | 2017-04-20 | 3.5 | BM | ST58 | B1 | blaCTX.M.3 |
| SAMN27150402 | 22 | 2017-04-27 | 3.4 | BM | ST57 | E | blaCTX.M.2 |
| SAMN27150403 | 23 | 2017-05-08 | 3.3 | BM | ST162 | B1 | blaCTX.M.65 |
| SAMN27150404 | 25 | 2017-05-20 | 3.5 | BM | ST2076 | D | new |
| SAMN27150405 | 26 | 2017-05-02 | 3.4 | BM | ST354 | F | blaCTX.M.3 |
| SAMN27150406 | 28 | 2017-06-01 | 4.0 | BM | ST38 | D | blaCTX.M.14 |
| SAMN27150407 | 29 | 2017-05-09 | 3.0 | BM | ST162 | B1 | blaCTX.M.65 |
| SAMN27150408 | 30 | 2017-05-30 | 3.3 | BM | ST38 | D | blaCTX.M.27 |
| SAMN27150409 | 31 | 2017-05-03 | 2.8 | BM | ST394 | D | blaCTX.M.15 |
| SAMN27150410 | 34 | 2017-05-22 | 2.9 | BM | ST648 | F | blaCTX.M.2 |
| SAMN27150411 | 35 | 2017-08-01 | 5.5 | BM | ST350 | E | blaCTX.M.2 |
| SAMN27150412 | 36 | 2017-08-14 | 5.1 | BM | ST12517 | B2 | blaCTX.M.15 |
| SAMN27150413 | 37 | 2017-09-05 | 5.9 | BM | ST155 | B1 | blaCTX.M.55 |
| SAMN27150414 | 38 | 2017-05-22 | 2.6 | BM | ST449 | D | blaCTX.M.55 |
| SAMN27150415 | 41 | 2017-10-02 | 5.8 | BM | ST1716 | A | blaCTX.M.65 |
| SAMN27150416 | 44 | 2017-10-27 | 6.0 | BM | ST131 | B2 | blaCTX.M.15 |
| SAMN27150417 | 46 | 2017-08-21 | 2.9 | BM | ST617 | A | blaCTX.M.14, blaCTX.M.55 |
| SAMN27150418 | 1 | 2017-10-31 | 12.5 | Food | ST162 | B1 | blaCTX.M.65 |
| SAMN27150419 | 2 | 2017-10-13 | 10.8 | Food | ST48 | A | blaCTX.M.65 |
| SAMN27150420 | 3 | 2017-12-05 | 12.6 | Food | ST58 | B1 | blaCTX.M.65 |
| SAMN27150421 | 4 | 2017-09-18 | 9.4 | Food | ST457 | F | blaCTX.M.27 |
| SAMN27150422 | 5 | 2017-08-21 | 8.8 | Food | ST12394 | A | blaCTX.M.15 |
| SAMN27150423 | 6 | 2017-10-15 | 10.5 | Food | ST1137 | A | blaCTX.M.55 |
| SAMN27150424 | 7 | 2017-12-11 | 12.0 | Food | ST354 | F | blaCTX.M.3 |
| SAMN27150425 | 8 | 2017-08-21 | 8.5 | Food | ST1485 | F | blaCTX.M.65 |
| SAMN27150426 | 9 | 2017-12-11 | 12.2 | Food | ST1193 | B2 | blaCTX.M.27 |
| SAMN27150427 | 10 | 2017-12-06 | 11.9 | Food | ST10 | A | blaCTX.M.55 |
| SAMN27150428 | 11 | 2017-09-18 | 9.0 | Food | ST744 | A | blaCTX.M.55 |
| SAMN27150429 | 12 | 2017-12-04 | 11.8 | Food | ST617 | A | blaCTX.M.14 |
| SAMN27150430 | 13 | 2017-09-18 | 8.9 | Food | ST10 | A | blaCTX.M.65 |
| SAMN27150431 | 14 | 2017-10-03 | 9.1 | Food | ST162 | B1 | blaCTX.M.65 |
| SAMN27150432 | 15 | 2017-09-13 | 8.6 | Food | ST2732 | D | blaCTX.M.55 |
| SAMN27150433 | 16 | 2017-09-18 | 9.2 | Food | ST44 | A | blaCTX.M.15 |
| SAMN27150434 | 17 | 2018-01-23 | 13.2 | Food | ST227 | A | blaCTX.M.55, blaCTX.M.65 |
| SAMN27150435 | 18 | 2017-10-16 | 9.3 | Food | ST617 | A | blaCTX.M.3 |
| SAMN27150436 | 19 | 2017-10-10 | 8.9 | Food | ST648 | F | blaCTX.M.14 |
| SAMN27150437 | 20 | 2018-01-02 | 11.9 | Food | ST117 | G | blaCTX.M.55 |
| SAMN27150438 | 21 | 2017-10-16 | 8.9 | Food | ST131 | B2 | blaCTX.M.15 |
| SAMN27150439 | 22 | 2017-10-25 | 9.3 | Food | ST457 | F | blaCTX.M.27 |
| SAMN27150440 | 23 | 2017-11-08 | 9.3 | Food | ST354 | F | blaCTX.M.55 |
| SAMN27150441 | 24 | 2018-01-02 | 11.0 | Food | ST617 | A | blaCTX.M.3 |
| SAMN27150442 | 25 | 2017-11-14 | 9.3 | Food | ST617 | A | blaCTX.M.14 |
| SAMN27150443 | 26 | 2018-01-03 | 11.4 | Food | ST744 | A | blaCTX.M.55 |
| SAMN27150444 | 27 | 2017-10-23 | 8.7 | Food | ST162 | B1 | blaCTX.M.55 |
| SAMN27150445 | 28 | 2018-02-07 | 12.2 | Food | ST609 | A | blaCTX.M.55 |
| SAMN27150446 | 29 | 2018-02-16 | 12.2 | Food | ST617 | A | blaCTX.M.3 |
| SAMN27150447 | 30 | 2017-12-05 | 9.4 | Food | ST1193 | B2 | blaCTX.M.27 |
| SAMN27150448 | 31 | 2018-02-06 | 11.9 | Food | ST131 | B2 | blaSHV.12 |
| SAMN27150449 | 32 | 2017-11-08 | 8.5 | Food | ST10 | A | blaCTX.M.65 |
| SAMN27150450 | 33 | 2018-04-17 | 13.5 | Food | ST12395 | B1 | blaCTX.M.55 |
| SAMN27150451 | 34 | 2017-11-20 | 8.8 | Food | ST449 | D | blaCTX.M.55 |
| SAMN27150452 | 35 | 2018-02-05 | 11.7 | Food | ST1196 | B1 | blaCTX.M.55 |
| SAMN27150453 | 36 | 2018-03-20 | 12.3 | Food | ST617 | A | blaCTX.M.15 |
| SAMN27150454 | 37 | 2018-03-05 | 11.9 | Food | ST58 | B1 | blaCTX.M.2 |
| SAMN27150455 | 38 | 2017-11-20 | 8.5 | Food | ST227 | A | blaCTX.M.3 |
| SAMN27150456 | 39 | 2017-12-26 | 9.2 | Food | ST354 | F | blaCTX.M.55 |
| SAMN27150457 | 40 | 2017-12-06 | 8.7 | Food | ST617 | A | blaCTX.M.14 |
| SAMN27150458 | 41 | 2018-04-03 | 11.9 | Food | ST38 | D | blaCTX.M.14 |
| SAMN27150459 | 42 | 2018-02-27 | 10.5 | Food | ST617 | A | blaCTX.M.55 |
| SAMN27150460 | 43 | 2018-08-06 | 15.6 | Food | ST752 | A | blaCTX.M.55 |
| SAMN27150461 | 44 | 2018-04-25 | 11.9 | Food | ST38 | D | blaCTX.M.14b |
| SAMN27150462 | 45 | 2018-04-24 | 11.8 | Food | ST43 | A | blaCTX.M.3 |
| SAMN27150463 | 46 | 2018-09-24 | 16.0 | Food | ST10 | A | blaCTX.M.65 |
| SAMN27150464 | 47 | 2018-05-22 | 11.9 | Food | ST38 | D | blaCTX.M.14 |

*Note*: BM= only received breast milk for ≥90% of the past 30 days. Food=consumed complementary foods for 100% of the past 60 days while also receiving breastmilk.

**Figure S1**. Directed acyclic graph for the association between breastfeeding and incident gut colonization with ESBL-producing Enterobacterales.

*SES=Socioeconomic status.

**Figure S2**. Participant flow chart outlining the previously collected samples and paired metadata included in this analysis.
